# Regressive vision transformer for dog cardiomegaly assessment

**DOI:** 10.1038/s41598-023-50063-x

**Published:** 2024-01-17

**Authors:** Jialu Li, Youshan Zhang

**Affiliations:** 1 Master of Public Administration, Cornell University, Ithaca, NY 14853 USA; 2 Computer Science and Artificial Intelligence, Yeshiva University, New York, NY 10033 USA

**Keywords:** Biomedical engineering, Cardiovascular biology

## Abstract

Cardiac disease is one of the leading causes of death in dogs. Automatic cardiomegaly detection has great significance in helping clinicians improve the accuracy of the diagnosis process. Deep learning methods show promising results in improving cardiomegaly classification accuracy, while they are still not widely applied in clinical trials due to the difficulty in mapping predicted results with input radiographs. To overcome these challenges, we first collect large-scale dog heart X-ray images. We then develop a dog heart labeling tool and apply a few-shot generalization strategy to accelerate the label speed. We also develop a regressive vision transformer model with an orthogonal layer to bridge traditional clinically used VHS metric with deep learning models. Extensive experimental results demonstrate that the proposed model achieves state-of-the-art performance.

## Introduction

Pet health has gained increasing attention in recent years. Deep learning techniques have demonstrated their superiority in processing medical image data and their profound impact on providing animal health services. With the development of convolutional neural networks (CNN), radiologists autonomously identify complicated patterns with computer vision algorithms that are accurate for all imaging modalities. Since most degenerative canine heart diseases accompany cardiomegaly, early detection of cardiac enlargement is a priority healthcare issue for dogs^1^. Applying AI technologies to dog cardiomegaly assessment can not only reduce the time and costs involved in pet diseases diagnosis and treatment, but also expand their use in the less AI-focused veterinary medicine field, compared to human medicine^2^. There are also existing publications that focus on the diagnostics of dogs for different diseases such as cardiomegaly detection^3,4^, atrial enlargement^5^, cardiogenic pulmonary edema^6^, bone fractures detection^7^, etc. However, most of these applications are related to the image classification of different diseases. Deep learning models output the classification labels given the input of radiographs. For clinicians, the output is not reliable since it is difficult to understand the reason why the deep network can obtain these labels. Although some models show the heatmap of classification results^3^, these heatmaps are still error-prone and do not show the correct decision areas that clinicians use for diagnosing. Therefore, it is necessary to develop models to help clinicians better understand radiographs that can be applied to animal medical images.

However, critical challenges remain, that is, to identify a useful bridge that connects deep learning methods and clinical trials. Clinicians who have less background in deep learning (DL) still do not trust the results from DL methods, even if they can achieve high performance. These DL results lack the explanation of original images and are not easy to map the predictions with input images. Therefore, it is essential to identify metrics that are frequently used by clinicians.

One useful method for clinicians to diagnose heart enlargement is to calculate the vertebral heart scale (VHS). If VHS is larger than a threshold, it will be diagnosed as an abnormal heart. While this method is error-prone and inefficient since the key points of the VHS score are manually determined (time-consuming), and different clinicians can have different estimations of these points. Especially, it is challenging to ensure the correctness and perpendicularity of these points. A similar method for clinicians to asses human cardiomegaly is to measure the cardiothoracic ratio (CTR) score. However, the aforementioned two challenges still exist. Therefore, we need to develop DL methods to overcome these two issues. In addition, it will be useful to provide an initial diagnosis to help clinicians accelerate the diagnosis speed.

More broadly, automatic cardiomegaly detection is not only useful for the diagnosis of clinicians and doctors, but also beneficial for institutions (including industrial and academic) to develop tools to assist the diagnosis process. The DL methods are still not trusted by clinicians, which indicates that there is still a gap between advanced DL methods and traditional diagnosis methods. Clinicians still waste time manually estimating cardiomegaly, while engineers are pursuing better DL models for diagnosis, which are not widely applied in clinical trials.

To overcome the aforementioned challenges, our contributions are threefold:

- We present a benchmark DogHeart dataset with the goal of advancing the state-of-the-art in dog cardiomegaly assessment.
- We propose a regressive vision transformer model to predict the VHS score and design an orthogonal layer to ensure the perpendicularity between the long and short axes of the heart area.
- We also develop a dog heart analysis tool to label the collected dataset and apply a few-shot generalization strategy to accelerate the data label process.

## Related work

**Figure 1:**
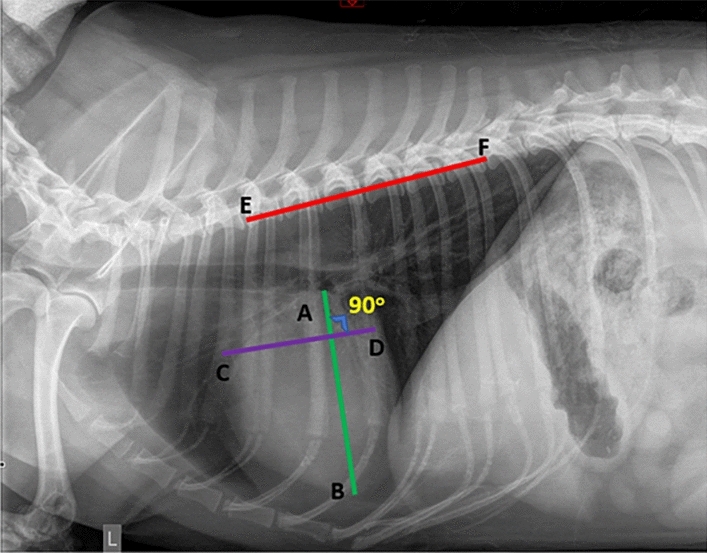
Our calculation of VHS, there are six key points (A, B, C, D, E and F). VHS = 6 × (AB + CD)/EF.

Thoracic radiographs are one of the most frequently used tools to diagnose cardiac disease. There are three steps to calculate the traditional VHS score. Firstly, we need to calculate the short (S) and long (L) axes of the dog’s heart area. Secondly, we need to identify the position of the fourth vertebral body of the spine. Lastly, we could calculate the VHS score using the sum of the long and short axis length divided by the vertebral length. Figure 1 shows how we calculate the VHS score. Many studies explored the application of VHS in diagnosing cardiomegaly on the size of a dog heart^8–10^. Rungpupradit et al.^11^ compared the conventional and applied VHS methods in healthy Thai domestic shorthair cats with abnormal thoracic vertebrae. The applied VHS methods reduce the limitation of conventional VHS methods by using the lateral view axis sum divided by the length of each thoracic vertebra. Tan et al.^12^ retrospectively evaluated Modified Radiographic Chest Volume (mRCV) and VHS for correlation with pulmonary patterns in dogs. The paper found that there are wide variations in VHS, and they are significantly associated with pulmonary patterns. Bappah et al.^13^ evaluated the relationship between VHS and cardiac sphericity and discovered that there is a strong correlation between VHS and cardiac sphericity index in dogs. However, these manually labeled VHS is error-prone and time-consuming.

### CNN models

Deep learning approaches are later introduced to assist the VHS method in veterinary medicine for diagnosing canine cardiomegaly. Zhang et al.^8^ calculated the concrete value of VHS with the relative position of 16 key points detected by the deep learning model and combined the results with the VHS reference range of all dog breeds to assist in the evaluation of the canine cardiomegaly. Jeong and Sung^1^ developed a new deep learning-based radiographic index, “adjusted heart volume index” (aHVI), quantifying canine heart size using retrospective data for dog diagnosis. Burti et al.^3^ developed a computer-aided detection (CAD) device based on convolutional neural networks (CNNs) to detect cardiomegaly from plain radiographs in dogs. Dumortier et al.^14^ developed a CNN based on ResNet50V2 to assess its performance in classifying feline Thoracic radiograph (TR) images in cats with or without Radiographic Pulmonary Patterns (RPPs) and to propose an optimized framework for better performance. Müller et al.^15^ proposed an AI algorithm to detect pleural effusion in thoracic radiographs of dogs. However, automatic traditional clinicians frequently used VHS calculation is not yet well-explored. One recent work^16^ estimated the VHS by using one CNN model, and they showed a consistent result between their model and two specialists, but their model’s architecture is unclear, and the process of calculating VHS is unclear. Therefore, we develop a regressive model to exactly determine the long and short axes of the canine heart and the vertebrate positions.

### Vision transformer methods

In recent years, vision transformer (ViT) began to dominate in image classification tasks, and has shown promising performance compared to state-of-the-art convolutional networks^17^. ViT models have also been explored in the medical imaging field. Yu et al.^18^ applied ViT for the retinal disease classification tasks by pre-training the MIL-VT model on a large fundus image database and fine-tuning on downstream retinal disease classification tasks. The model outperformed CNN models. Gao et al.^19^ intended to compare the performance of ViT based on attention models and DenseNet based on CNN on the predicted diagnosis of the COVID-19 virus from chest radiographs. The initial results showed that ViT performed better than DenseNet. Gheflati et al.^20^ utilized ViT to classify breast US images using different augmentation strategies and adopted a weighted cross-entropy loss function to deal with the potential imbalance in breast ultrasound datasets. Results indicated that ViT models are comparable to or even better than CNNs in the classification of US breast images. However, ViT has not been widely adopted in the veterinary medicine field, for example, in the detection of dog cardiomegaly, which is one of the predominant dog diseases. We are the first to apply a vision transformer-based method to this area.

## Methods

### Motivation

VHS^21^ has been used as one of the standard methods to evaluate cardiac silhouette size on thoracic radiographs for animals. However, there are two major issues with the calculation of VHS. (1). The estimation of long and short axes positions is error-prone. Different clinicians can give different estimations of the positions. (2). The VHS score is only estimated in one decimal point, which is not accurate. Existing deep learning methods often treat the detection of cardiomegaly as an image classification problem^3,4,8^. Although some methods achieved reasonable accuracy, they still did not widely use in clinical trials. The key reason is that the deep classification model only outputs the final classification result (cardiomegaly or non-cardiomegaly) with its probability, most clinicians who have no background in deep learning or machine learning models will not trust the results. Some works that visualize the decision of deep learning models with gradient reversed-based methods (e.g., Grad-CAM^22^) still did not output the correct decision boundary image of the X-ray images. Therefore, it is necessary to develop a technique that combines the traditional and deep-learning models to improve the accuracy and ease the interpretation by clinicians who have a limited background in deep learning.

**Figure 2:**
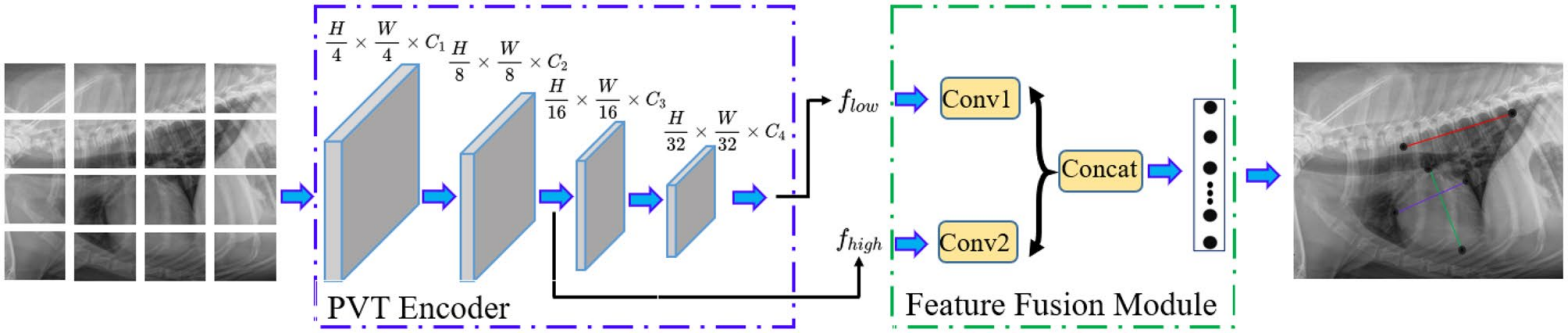
The overall architecture of our proposed regressive vision transformer (RVT) model. We first employ a pyramid vision transformer as an encoder to extract low and high-level features. Then, we add a feature fusion module to predict six key points of the VHS score. An orthogonal layer is proposed to ensure the perpendicularity between line segments AB and CD as shown in Fig. 1.

### Problem

Given dog X-ray images $X = \{x_i\}_{i=1}^{n}$, we aim to accurately diagnose whether there is cardiomegaly in these images. We convert it into three category classification problem (small, normal and large). To reduce the uncertainty of predicted results, we directly output the six key points of clinical frequently used VHS score, which can be easily interpreted by clinicians. Given the dog X-ray images with its labeled key points $P = \{y_i\}_{i=1}^{n}$, we propose to minimize the error between predictions of any model *f*(*X*) and *P*, and get high diagnosis accuracy.

### PVT-transformer block

Because of the traditional transformers’ single-scale low-resolution representations, it is difficult for vision transformer models to implement dense prediction tasks and effectively leverage the rich transformer layers in the encoder for excavating helpful multi-modal context. In addition, due to the global self-attention mechanism, this method incurs high computational and memory costs. To alleviate this problem, the PVT transformer is designed^23^. The key design feature of the PVT transformer is to design a progressive shrinking pyramid and spatial-reduction attention (SRA). It is built by designing a module based on SRA as a substitute for a multi-head self-attention (MSA) module in the transformer block. Each PVT transformer block is composed of an attention layer and a feed-forward layer, including a LayerNorm (LN) layer, a two-layer MLP, and GELU nonlinearity. The SRA module is applied in series on the transformer block. With such an SRA module attention scheme, consecutive PVT transformer blocks are formulated as:

1 $$\begin{aligned}SRA(Q,K,V)&=Concat(head_0, \ldots ,head_{N_i})W^O, \end{aligned}$$

2 $$\begin{aligned} head_j&=Attention(QW^{Q}_{j},SR(K)W^K_j,SR(V)W^V_j), \end{aligned}$$

where Concat($\cdot$) is the concatenation operation. $W_j^Q,W_j^K,W_j^V \in R^{C_i \times d_head}$ and $W^O \in R^{C_i \times C_i}$ are linear projection parameters. $N_i$ is the head number of the attention layer in stage *i*. $SR(\cdot )$ is the spatial dimension reduction of the input sequence operation, which is defined as:

3 $$\begin{aligned} SR(x)=Norm(Reshape(x,R_i)W^S), \end{aligned}$$

where $x \in R^{(H_{i}W_i)\times C_i}$ is a input sequence, and $R_i$ is the reduction ratio of the attention layers in stage *i*. $W_S\in R^{(R_{i}^{2}C_i)\times C_i}$ is a linear projection to reduce the dimension of the input sequence to $C_i$. Norm($\cdot$) is the layer normalization. The self-attention is computed according to:

4 $$\begin{aligned} Attention(Q,K,V)=SoftMax\left(\frac{QK^T}{\sqrt{d}} +B \right)V, \end{aligned}$$

where $Q,K,V\in R^{M^2\times d}$ are the query, key and value matrices; d is the query/key dimension, and $M^2$ is the number of patches in a window and and *B* is taken from bias matrix $\hat{B} \in \mathbb {R}^{{(2M-1)\times (2M+1)}}$.

### Feature fusion module

We can extract low-level features ($f_{low}$) and high-level features ($f_{high}$) from the PVT encoder. Low-level features can extract rich detail information, such as texture, color, and edges, while high-level features can extract objects and larger shapes. Therefore, we develop a feature fusion module (FFM) to fuse these two different features to extract robust features. Specifically, we propose to use convolutional layers to fuse these two features, as shown in Fig. 2. Conv1 is a convolutional unit composed of $1 \times 16$ with padding set to 1, and stride size to 96. Conv2 has a convolutional unit composed of $1 \times 16$ with padding set to 1, stride size to 16, and dilation size of 9. We can get the fused features as in Eq. (5).

5 $$\begin{aligned} F_F = f_{low} \odot f_{low}, \end{aligned}$$

where $F_F$ means the fused features, and $\odot$ is the feature concatenation function.

### Orthogonal layer

To calculate the VHS score, we need to guarantee the perpendicularity between line segments AB and CD, as shown in Fig. 1. Hence, we develop an orthogonal layer to ensure the perpendicularity between them. The final fully connected layer has twelve units as the output, corresponding to six different points. In the orthogonal layer, we will check the perpendicularity of the first four points (eight numbers), since the last 2 points refer to the length of six dog vertebrae. Given $(x_1, y_1), (x_2, y_2), (x_3, y_3)$ and $(x_4, y_4)$, we will use $\hat{y_4}$ to replace original $y_4$ as follows:

6 $$\begin{aligned} s = - \frac{x_1-x_2}{y_1-y_2}, \quad \hat{y_4} = s (x_4 - x_3) + y_3, \end{aligned}$$

where *s* is the slop of the line segment CD. Therefore, we can ensure the perpendicularity in our orthogonal layer and get a better estimation of VHS score.

### Objective function

In our dog cardiomegaly assessment, we not only aim to estimate the six different key points, but we want to get correct diagnosis results. As mentioned in Fig. 1, the VHS can be calculated by VHS = 6 $\times$ (AB + CD)/EF. Hence, we could define different categories of dog cardiomegaly conditions as follows (we get optimal thresholds 8.2 and 10 based on the accuracy from validation datasets).

7 $$\begin{aligned} y_t = {\left\{ \begin{array}{ll} 0 &{} \text {VHS }< \text { 8.2}\\ 1 &{} \text {(VHS }\ge \text { 8.2)} \& \text { ( VHS }\le \text { 10)},\\ 2 &{} \text {Otherwise} \end{array}\right. } \end{aligned}$$

We can minimize cross-entropy loss to improve the accuracy of diagnosis and minimize mean square error to enhance the closeness of six key points between prediction and ground truth. Fig. 2 depicts the overall framework of our RVT model. Considering all components, our model minimizes the following objective function:

8 $$\begin{aligned} \mathscr {L} = \frac{1}{n} \sum _{i=1}^{n} \{\mathscr {L}_{ce} (f(x_i), y_t^i) + \gamma \mathscr {L}_{MSE} (f(x_i), y_i) \}, \end{aligned}$$

where $\mathscr {L}_{ce}$ is the typical cross-entropy loss, $\mathscr {L}_{MSE}$ is the mean-square-error (MSE) loss and *f* is our RVT model. $\mathscr {L}_{ce}$ can minimize the three classes difference, while $\mathscr {L}_{MSE}$ can minimize six points difference between the prediction and ground truth. $\gamma$ is the balance factor. The overall training algorithm is shown in Algorithm 1.

**Algorithm 1:**
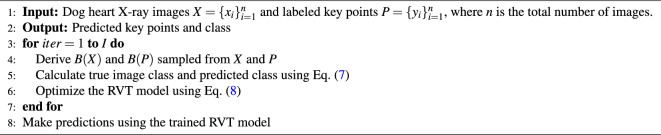
Regressive Vision Transformer (RVT). $B(\cdot )$ denotes the mini-batch training sets, and *I* is the number of iterations.

## Datasets

### Data collection

**Figure 3:**
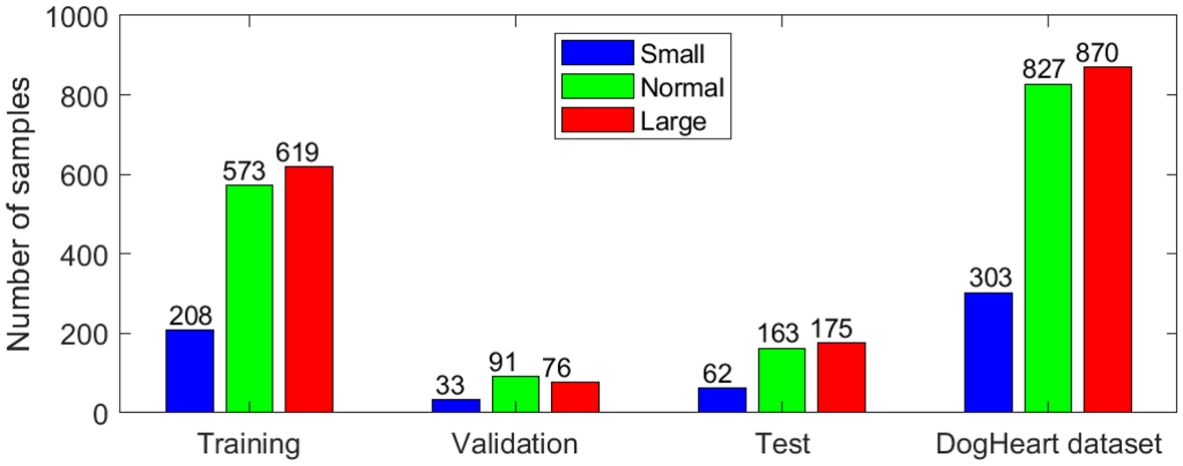
Data distribution of training, validation, test, and all DogHeart dataset, respectively.

**Figure 4:**
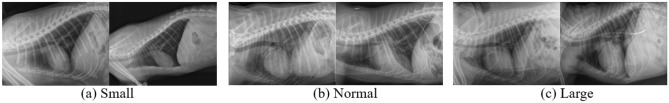
Six sample dog X-ray images of our collected DogHeart dataset.

Our dog's X-ray images were collected from Shanghai Aichong Pet Hospital (Fig. 3). All X-ray images were cropped when received, without any private information, therefore, the research would not violate the privacy of dogs or their owners. A total of 6389 canine thoracic radiographs were retrieved, and 1400 images were selected as the training, 200 images were included in the validation dataset, and 400 images were included as the test dataset. Table 1 shows the statistics of each category (small, normal, and large) in our DogHeart dataset. We show two sample images of each category in Fig. 4. Our DogHeart dataset has 2000 valid images in total. There are 1400 images (70%) in the training dataset, 200 images (10%) in the validation dataset, and 400 images (20%) in the test dataset. Each image corresponds to an individual dog. All images with VHS scores below 8.2 are classified as small hearts, normal dogs are between 8.2 and 10, and large dogs are above 10. Table 1 and Fig. 3 show that there are fewer samples of the small dog category, and the number of normal and large dog categories are balanced in our collected DogHeart dataset.

**Table 1:** Statistics on DogHeart dataset.

Datasets	Training	Validation	Test
# of Small	208	33	62
# of Normal	573	91	163
# of Large	619	76	175
Total #	1400	200	400

### Data labeling

To accurately measure the VHS score, we need to provide the ground truth of six key points in the dog X-ray images. For any machine learning algorithms, we have to provide enough labeled datasets to achieve a good performance. However, the data labeling process is time-consuming, tedious, and expensive. Given that there is no specific software for dog heart VHS labeling, we developed a dog heart analysis software. This software has three key functions. (1). It can open a dog X-ray image and label it to create six key points and show the overlapping between the raw X-ray image and labeled points. (2). We can save created points. The software also supports human verification. All accepted points will be saved in a ‘Saved’ folder. (3). We can also compare the ground truth points with predicted points from any deep learning models to validate the performance of the models. More details about our developed dog heart analysis software can be found in the supplementary Fig. 1. To get the VHS score, we first label the four key points of the dog heart area. For the long axis of the heart, the rule is to draw a line from the carina to the apex of the heart at its most ventral point. For the short axis of the heart, we need to draw a line from the widest point, and it should be perpendicular to the long axis. Our software can automatically adjust for the perpendicularity between the two axes. Then, we draw a line that starts at the cranial aspect of the fourth thoracic vertebrae (T4) and ends with the ninth vertebrate.

**Table 2:** Results comparisons of different methods in validation and test datasets (accuracy is multiplied by 100).

Networks	Validation		Test	
	C_Accuracy	R_Accuracy	C_Accuracy	R_Accuracy
GoogleNet^24^	75.0	77.5	73.8	74.8
VGG16^25^	77.5	78.5	74.8	75.0
ResNet50^26^	77.5	80.0	75.3	78.3
DenseNet201^27^	77.5	77.0	75.5	80.8
Inceptionv3^28^	75.0	79.0	78.0	80.0
Xception^29^	75.0	78.5	73.0	75.3
InceptionResnetV2^30^	76.5	77.5	75.5	78.8
NasnetLarge^31^	79.5	80.0	78.8	82.5
EfficientNetB7^32^	79.5	82.0	77.5	84.5
Vision transformer^17^	77.5	80.0	73.3	77.5
CONVT^33^	80.0	82.0	75.3	85.3
Beit_large^34^	70.5	71.0	64.0	74.3
RVT	82.5	85.0	82.8	87.3

**Table 3:** Methods information comparisons [MB: megabyte; M: million; S: seconds per image (A6000 GPU time)].

Datasets	Depth	Size (MB)	Parameters (M)	Training time (S)	Inference time (S)
GoogleNet^24^	22	27	5.631	0.0643	0.0433
VGG16^25^	16	528	134.310	0.0857	0.0500
ResNet50^26^	50	96	23.004	0.0700	0.0450
DenseNet201^27^	201	77	20.037	0.0986	0.0550
Inceptionv3^28^	48	89	24.377	0.8038	0.0513
Xception^29^	71	85	37.916	0.0957	0.0517
InceptionResnetV2^30^	164	209	54.325	0.1029	0.0533
NasnetLarge^31^	533	332	84.769	1.7736	0.4317
EfficientNetB7^32^	438	256	63.818	2.7429	0.5083
Vision transformer^17^	225	327.366	85.817M	0.1271	0.0817
CONVT^33^	208	327.226	85.780	0.0950	0.0667
Beit_large^34^	369	1354.662	304.662	7.7307	2.2033
RVT	340	74.965	19.626	4.3943	0.9200

The inference time includes both validation and test datasets). Note that parameters are trainable parameters using our dataset, and they will be different from the number of parameters in the original model.

#### Few-shot generalization

Although we developed a specific dog heart analysis software, it takes around 5 min to label one X-ray image. We have 2000 images, and it is still time-consuming to label all images. To accelerate the labeling process, we propose to utilize the few-shot generation to first predict coarse points for X-ray images. Then we can verify and update these coarse points to get better points. Few-shot learning aims to learn a robust model based on a few labeled samples, then improve the performance of new datasets. To ease the process of image labeling, we first manually labeled 150 X-ray images as training and 50 images as the test. We select ResNet50 as the prediction model and train the ResNet50 model using these 200 labeled images to get a basic model *f*. We then predict the coarse points via *f*(*I*). Given any unlabeled dog X-ray $x_i$, we can get all predicted coarse points as $\{f(x_i)\}_{i=1}^{n}$. Finally, these coarse points can be further modified using our developed dog heart analysis tool. After using the proposed few-shot generalization strategy, the whole dataset is labeled by two experts in 2 weeks. Each image is labeled by two experts. We calculate the intraclass correlation coefficient (ICC) of the labeled points from two experts. The ICC score is 0.952, which means that there is a high agreement between the labeled points of two human specialists.

## Experiments

To evaluate the performance of our proposed RVT model, we test it on our created DogHeart dataset, and compare it with 12 different state-of-the-art classification models, including GoogleNet^24^, VGG16^25^, ResNet50^26^, DenseNet201^27^, Inceptionv3^28^, Xception^29^, InceptionResnetV2^30^, NasnetLarge^31^, EfficientNetB7^32^, Vision transformer^17^, CONVT^33^, and Beit_large^34^. These 12 different models are trained on a benchmark ImageNet dataset. We omit some low-accuracy ImageNet models, e.g., AlexNet, and SqueezeNet. From GoogleNet to EfficientNetB7 are traditional convolution-based deep neural networks. From VT to Beit_large are vision transformer-based methods. Parameters in our RVT model are learning rate ($\epsilon = 3e^{-5}$), batch size (16), $\gamma = 0.01$, and the number of epochs (1000) are determined by performance on the validation datasets. Experiments are performed with an Adam optimizer on an RTX A6000 GPU. The input image size of the models is $[512 \times 512 \times 3]$. We use the Image function from the PIL library to convert an X-ray image into an RGB image ( “Image.open(img_path).convert(“RGB”)”, where img_path is the path of the X-ray image). There are 340 layers in our model, around 19.626 million trainable parameters, and the size of the model is 1852.52 megabytes. To evaluate the performance of all models, we report the accuracy of validation and test datasets to check whether these models could make a correct prediction of dog heart enlargement problem using $Accuracy = \sum _{i=1}^{n} (y_i == y_i^p)/n$, where $y_i$ is the true labels and $y_i^p$ is the predicted labels, and *n* is the total number of images in the dataset. All studies are conducted using the same training, validation, and test datasets.

**Figure 5:**
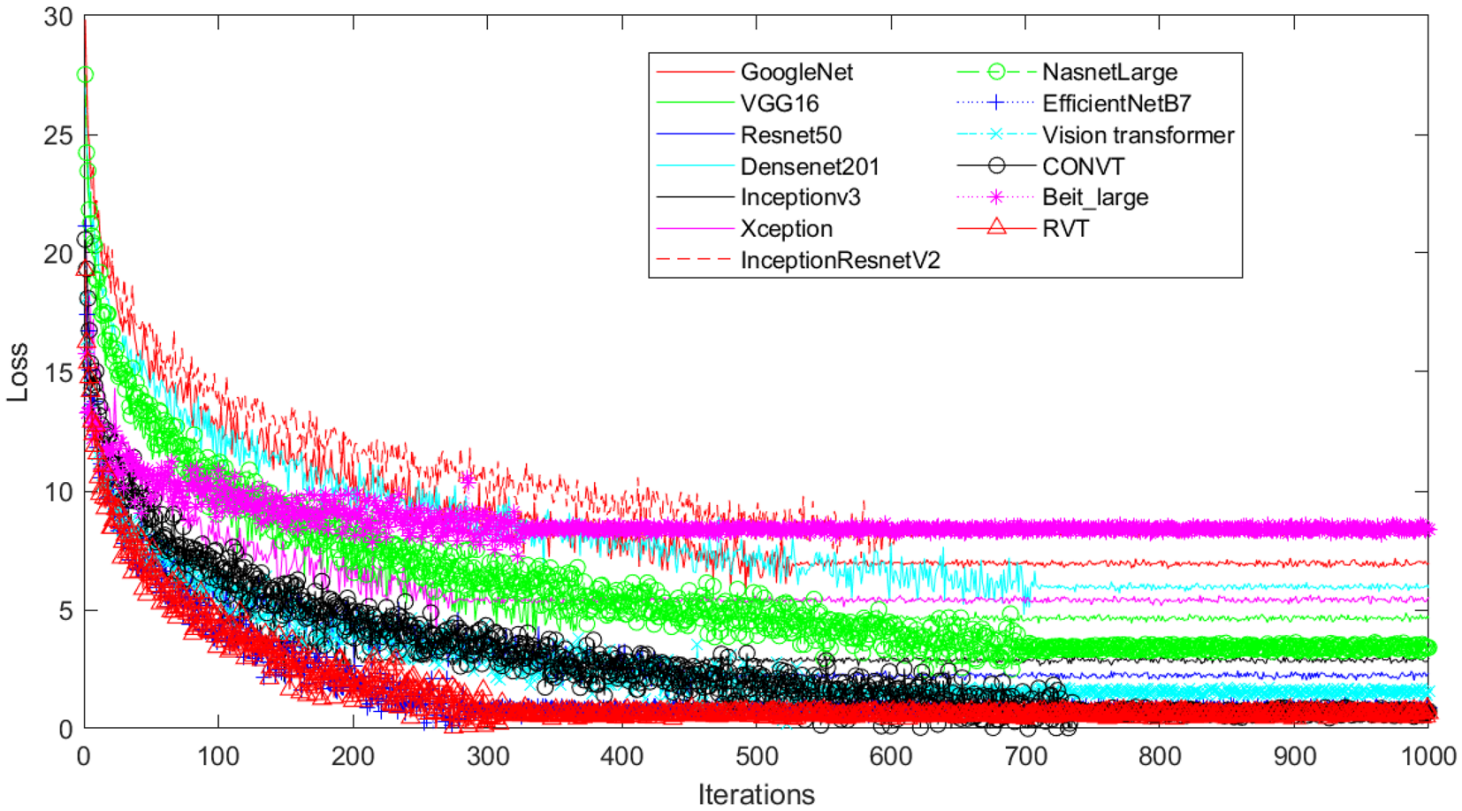
The training convergence of 13 different models.

As shown in Fig. 5, we use a large number of training iterations (1000) to ensure that all models are converged during the training. Densenet201, CONVT, and NasnetLarge models utilized more iterations to be converged. Beit_large, Xception, EfficientNetB7, and our proposal RVT utilized fewer iterations to be converged. In addition, the converged loss of the Beit_large model is around 8, which is bigger than other models. This also corresponds to results in Table 2 that the Beit_large model did not have a high accuracy. Similarly, the converged loss number of CONVT, EfficientNetB7, and our proposed RVT is close to 0, which implies that these three models have relatively high accuracy. Table 3 shows that deeper networks are more likely to utilize longer training and inference time. Among these 13 methods, Beit_large needs the longest training and inference time. Although our proposed RVT model uses less time than the Beit_large model, it needs more time than NasnetLarge and EfficientNetB7 models. This is due to more attention layers of Beit_large and our proposed RVT model. However, we need a more precise model, and 0.92 s is in the reasonable range. Therefore, our RVT model is suitable for dog cardiomegaly assessment.

**Figure 6:**
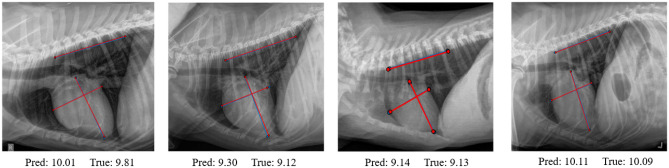
Predicted results of our RVT model. The red color lines are the ground truth lines and the blue color lines are the predicted results.

Figure 6 shows four predicted results using our RVT model, the predicted VHS scores are close to the ground truth, and the predicted lines are well aligned with the true lines.

**Figure 7:**
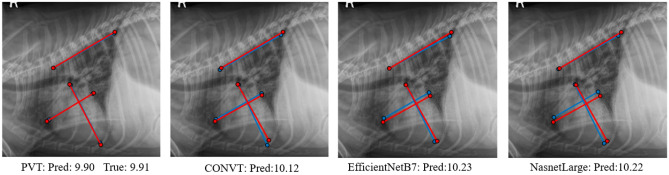
Predicted results comparison. The red color lines are the ground truth lines and the blue color lines are the predicted results.

We show the comparison results in Table 2. C_Accuracy means that the model is only trained based on cross-entropy loss, while R_Accuracy means that we train the model using the proposed loss function in Eq. (8). We can find that the accuracy of R_Accuracy is overall higher than C_Accuracy. Our proposed RVT model achieves the highest accuracy compared to other models in both C_Accuracy and R_Accuracy. We show four predicted results using our RVT model in Fig. 6. The predicted VHS scores and predicted lines are close to the ground truth. We also compare the predicted results of the three best baseline methods: NasnetLarge, EfficientNetB7, and CONVT models, as shown in Fig. 7. The predicted VHS and three lines of the RVT model are better than all three other models.

**Figure 8:**
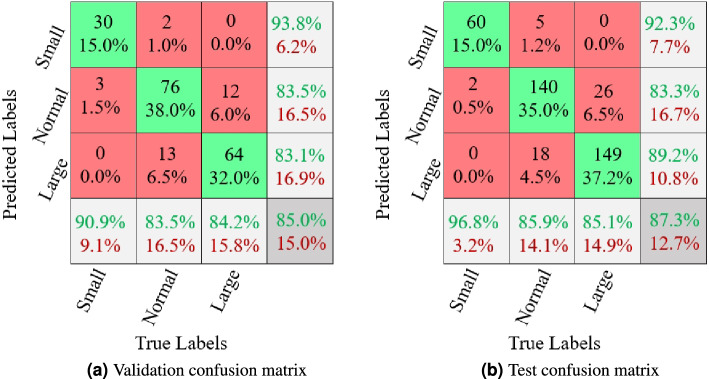
Confusion matrices of validation and test datasets. The X-axis is ground truth labels, and the Y-axis is predicted labels. The numbers (*e*.*g*.,  30, 2, 0) are the predicted class numbers. Black percentages $e.g., 30/200 = 15\%$ are the ratio between the number of predicted labels and the total number of images. The green percentages are the correct predicted percentages, $e.g., 30/33 = 90.9\%$, and the red percentages are the wrong predicted percentages, $e.g., 3/33 = 9.1\%$.

From Table 2, we can observe that our joint loss function is better than single cross-entropy loss. We set the balance factor $\gamma = 0.01$ according to the performance of the validation dataset. In Fig. 8a, 30 out of 33 small images are predicted as small with a category accuracy of 90.9%, and it corresponds to 15% of the 200 samples. 3 out of 33 small images are predicted as normal with a wrong category accuracy of 9.1%, and it corresponds to 1.5% of the 200 samples. A similar explanation can be applied to the normal and large category images. From Fig. 8, we can find that the predicted labels for the small hearts category are higher than the other two classes in both validation and test datasets (90.9% and 96.8%). The predicted accuracies of normal and large categories are similar in both datasets (83.5% vs. 84.2% and 85.9% vs. 85.1%). We also list the results of AUC, precision, specificity, sensitivity of validation, and test datasets using our RVT model. From Table 4, we can also conclude that the model performance on the small heart category is better than the other two categories, which is because the small heart images are obviously different from normal and larger images. Meanwhile, the model’s performances on normal and large categories are similar across both validation and test datasets. From Table 2, in terms of R_accuracy, 8 over 13 methods have higher accuracy in the test dataset than in the validation dataset. However, in terms of C_accuracy, 11 out of 13 methods have higher accuracy in the validation dataset than in the test dataset. This implied that our proposed orthogonal layer could not only ensure the perpendicularity between the long and short axes of the heart area when calculating the VHS score but also further improve the accuracy, especially in improving the generalizability of test datasets.

In addition, we conduct an ablation study to show the effectiveness of three modules: PVT (P), feature fusion (F), and orthogonal layer (O) using the validation dataset in Table 5. Note that a PVT transformer is required for the experiment. We can observe that with more modules, our performance is improved, and feature fusion is more important than the orthogonal layer. From Fig. 7, we can find that the predicted key points of our RVT model are better than other models. Compared to Table 2 with Table 7, we can find that the pure accuracy of the PVT (P) model is better than all state-of-the-art models in the validation dataset, which reveals that our PVT performs better in dog cardiomegaly assessment. In addition, the inclusion of feature fusion (P+F) and orthogonal layer (P+O) achieve better accuracy than PVT alone, and further better than other models. We also conduct ablation studies in “Ablation study” section. Therefore, we can conclude that our proposed RVT model is suitable for dog cardiomegaly assessment.

**Table 4:** AUC, precision, specificity, sensitivity of validation and test datasets.

Metics	Validation			Test		
	Small	Normal	Large	Small	Normal	Large
AUC	0.9172	0.8294	0.8197	0.9274	0.8463	0.8390
Precision	0.9375	0.8352	0.8311	0.9231	0.8333	0.8922
Specificity	0.9880	0.8624	0.8952	0.9852	0.8819	0.9200
Sensitivity	0.9091	0.8352	0.8421	0.9677	0.8589	0.8514

**Table 5:** Ablation study on different modules on validation dataset.

Modules	P	P + F	P + O	P + F + O
RVT	82.5	84.0	83.5	85.0

**Table 6:** Results of different feature fusion layers.

Blocks	Blcok 1	Blcok 2	Blcok 3	Blcok 4
Accuracy	84.0	84.5	83.0	83.5

**Table 7:** Ablation study on different loss functions on test dataset.

	RVT	CONVT	EB7	NasnetLarge
Cross-entropy loss	82.3	80.0	77.0	77.3
Mean square error	83.5	81.3	78.3	78.0
All	84.8	82.0	79.5	79.5

**Table 8:** Ablation study of different methods on the orthogonal layer (accuracy is multiplied by 100. Bold text means better result).

Networks	Validation			Test		
	Original + O	Original	Improvement	Originall + O	Original	Improvement
GoogleNet^24^	**78.0**	77.5	0.5	**75.8**	74.8	1.0
VGG16^25^	**79.0**	78.5	0.5	**75.5**	75.0	0.5
ResNet50^26^	**80.5**	80.0	0.5	78.0	**78.5**	− 0.5
DenseNet201^27^	**77.5**	77.0	0.5	**81.8**	80.8	1.0
Inceptionv3^28^	**79.5**	79.0	0.5	**80.5**	80.0	0.5
Xception^29^	**79.0**	78.5	0.5	**75.8**	75.3	0.5
InceptionResnetV2^30^	**78.0**	77.5	0.5	**79.5**	78.5	1.0
NasnetLarge^31^	79.5	**80.0**	− 0.5	**83.8**	82.5	1.3
EfficientNetB7^32^	**82.5**	82.0	0.5	**85.5**	84.5	1.0
Vision transformer^17^	79.5	**80.0**	− 0.5	**79.5**	77.5	2.0
CONVT^33^	**82.5**	82.0	0.5	**87.5**	85.3	2.2
Beit_large^34^	**71.5**	71.0	0.5	**75.0**	74.3	0.7
**RVT**	**85.0**	84.0	1.0	**87.5**	85.3	2.2

**Figure 9:**
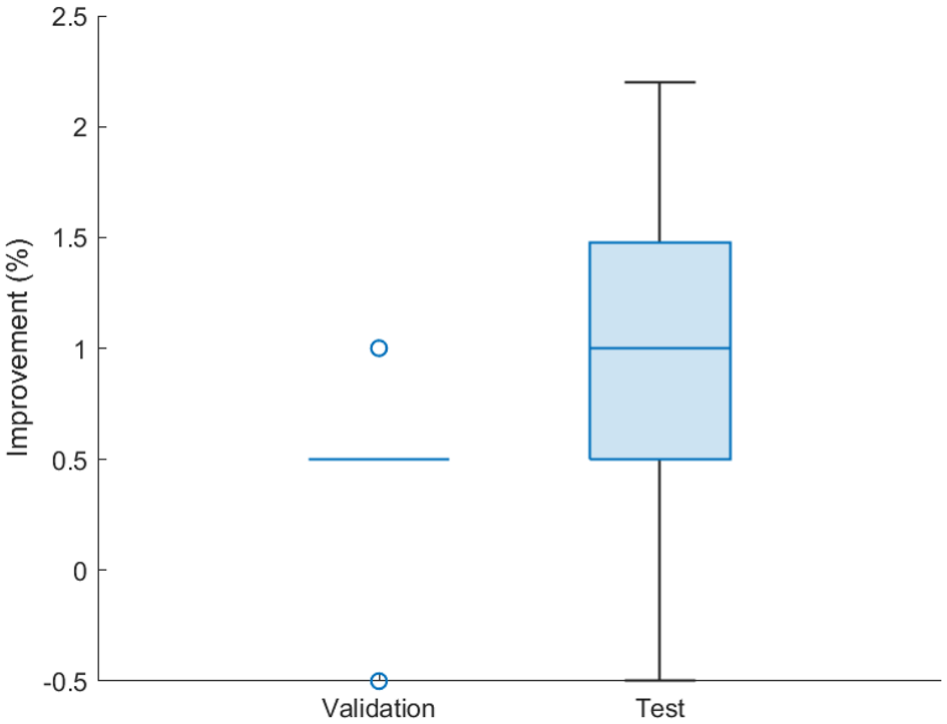
The boxplot of improvement after using the orthogonal layer.

## Ablation study

We first explore the effectiveness of different feature layers in Table 6. There are four blocks in our developed PVT encoder. We could extract features in each block. From Table 6, we could find that the second block has the best performance since we lost information in deep block 3 and block 4, and block 4 cannot extract enough features. Therefore, we extract high-level features from the second block. To explore which loss function is more useful in our DogHeart dataset, we conduct an ablation study in Table 7. We can find that the MSE loss function is more useful than the cross-entropy loss. One potential reason is that we calculate the accuracy based on the six key points. If the MSE loss is smaller, the predicted points are closer to the ground truth, and we can get higher accuracy. We show the robustness of our developed orthogonal layer on twelve baseline methods on both validation and test dataset in Table 8. The “original” of our PVT is the result of “P+F” in Table 5 of the main paper. We can find that applying the orthogonal layer (Original+O) improves the performance of most models. Therefore, we can conclude that the proposed orthogonal layer is effective in accurately predicting the location of six key points and improving classification accuracy. As shown in Table 8 and Fig. 9, the average improvement of the validation dataset is 0.385% (median improvement of 0.5%), and the average improvement of the test dataset is 1.03% (median improvement of 1%). Although the improvement is not significant, the orthogonal layer can still help us to increase the performance of cardiomegaly assessment. Most importantly, the orthogonal layer can maintain the perpendicularity between the long and short axes when we calculate VHS scores. Without the orthogonal layer, the predicted key points are not optimal, and clinicians will not trust these predictions. Therefore, our orthogonal layer is necessary for estimating VHS scores.

## Conclusion

In this paper, we propose a regressive vision transformer (RVT) model for dog cardiomegaly classification with a DogHeart dataset. We design an orthogonal layer to ensure the perpendicularity between the long and short axes of the heart area. In addition, we develop a dog heart analysis tool and propose to use few-shot generation to label all datasets. Extensive experimental results demonstrate that the proposed RVT model outperforms many state-of-the-art methods. Our proposed method is not limited to radiograph X-ray image diagnosis, but can be applied to other types of medical images, such as CT scans and ultrasounds. Our model can be extended to detect human cardiomegaly using different diagnosis technologies. In addition, clinicians can use our software for diagnosis even without expertise in deep learning. Therefore, our model has a number of broader impacts on different clinical diagnosis applications.

### Supplementary Information

Supplementary Figure 1.

## Data Availability

The source dataset and code will be available upon request. Please contact the corresponding author: Youshan Zhang, to access the data and code.
