# Supplementary material for: Regressive vision transformer for dog cardiomegaly assessment
Source: Sci Rep. 2024 Jan 17;14:1539. doi: 10.1038/s41598-023-50063-x (PMC10794204; doi:10.1038/s41598-023-50063-x)
Supplement: Supplementary file 1 — Supplementary Figure 1. [file 41598_2023_50063_MOESM1_ESM.pdf]

Supplementary Material: Regressive Vision Transformer for Dog Cardiomegaly Assessment

Jialu Li[1] **and Youshan Zhang**[2*]

[1]Cornell University, Master of Public Administration, Ithaca, NY, 14853, USA
[2]Yeshiva University, Computer Science and Artificial Intelligence, NYC, NY, 10033, USA
[*]youshan.zhang@yu.edu

ABSTRACT

The supplementary figure 1 shows the interface of our proposed dog heart analysis tool. This software is developed based on MATLAB 2022a. There are many supported functions in this tool. The order of the following explanation is from top to bottom and from left to right.

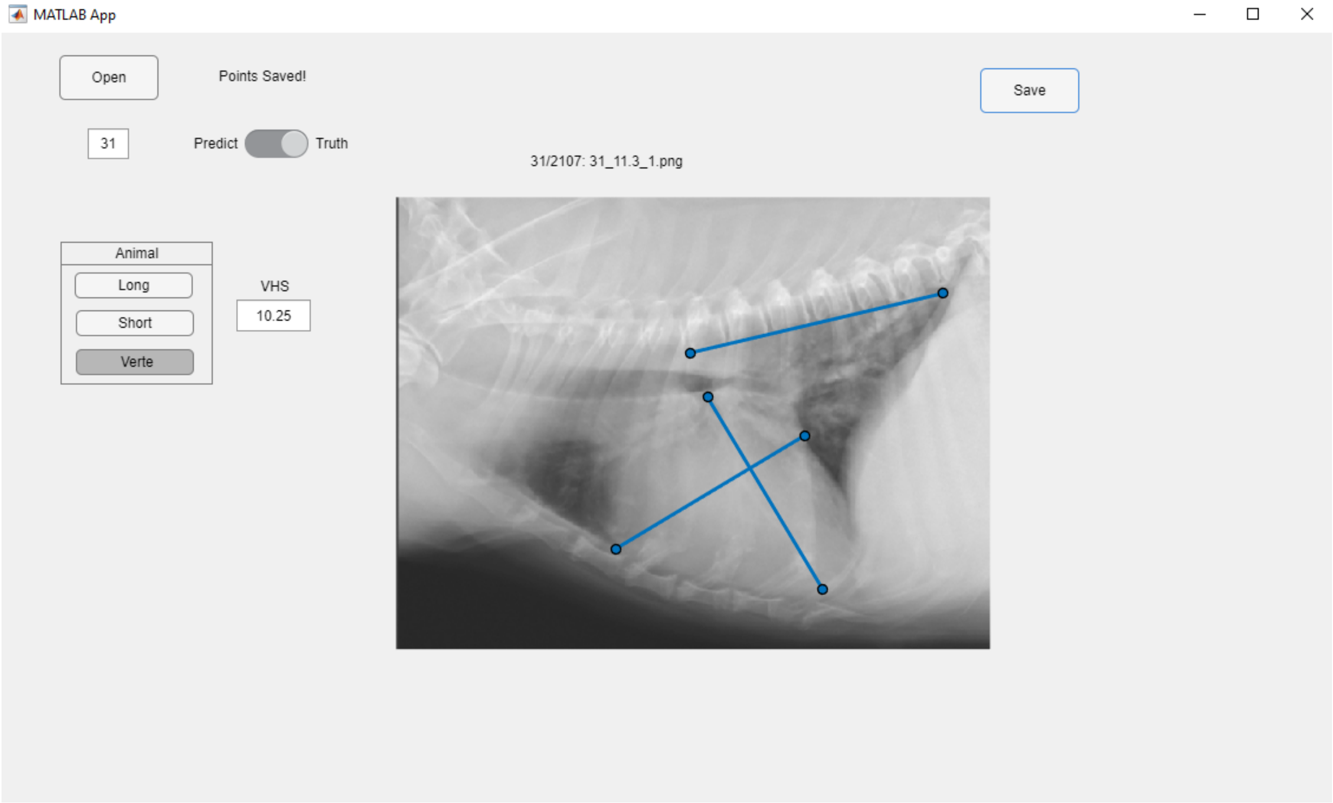

Supplementary figure 1. The interface of our developed dog heart analysis tool and an example of a labeled dog X-ray image. The blue color is the labeled lines. On the top of the image, "31/2107": 31 is the current dog X-ray image number, and 2107 is the total number of images. "31_11.3_1.png" is the image name. "Points saved!" will be displayed after clicking the "Save" button.

1. The "Open" button: we can select any dog X-ray image.
2. The edit text box: it will show the index number of the current image. "1" is the default initialization number. We can also directly change this number to jump to another image. The current number is 31 in the supplementary figure 1.

3. In the switch radio button box: it supports the switch between “Truth" and “Predict". The “Truth" is the labeled points, and the “Predict" is the predicted points from any model. Hence, we can compare the differences between human-labeled points and predicted labels from deep learning models.

4. In the select “Animal" box groups: once we click the “Long" button, we can draw the long axis of the heart area. We can also draw the short axis of the heart by clicking the “Short" button. If we click the “Verte", we can draw a line on the vertebrate, and it will automatically adjust the long and short axes and guarantee the perpendicularity between them.

5. The “VHS" text box: it will directly show the labeled VHS score. VHS = 10.25 in the supplementary figure 1.

6. Image information area: it will demonstrate the current image number, total image number, and image name, e.g., “31/2107:31_11.3_1.png”.

7. Image shown area: it will show the dog heart image with its drawn lines

8. The “Save" button, we can save the labeled six key points in a “Saved" folder.
